# TCL1 transgenic mouse model as a tool for the study of therapeutic targets and microenvironment in human B-cell chronic lymphocytic leukemia

**DOI:** 10.1038/cddis.2015.419

**Published:** 2016-01-28

**Authors:** A Bresin, L D'Abundo, M G Narducci, M T Fiorenza, C M Croce, M Negrini, G Russo

**Affiliations:** 1 Laboratorio di Oncologia Molecolare, Istituto Dermopatico dell'Immacolata, IDI-IRCCS, Rome, Italy; 2 Dipartimento di Morfologia, Chirurgia e Medicina Sperimentale, Università di Ferrara, Ferrara, Italy; 3 Dipartimento di Psicologia, Sezione di Neuroscienze, Università La Sapienza di Roma, Rome, Italy; 4 Human Cancer Genetics Program and Department of Molecular Virology, Immunology and Medical Genetics, OSU School of Medicine, Ohio State University, Columbus, OH, USA

## Abstract

Chronic lymphocytic leukemia (CLL) is a B-cell malignancy with a mature phenotype. In spite of its relatively indolent nature, no radical cure is as yet available. CLL is not associated with either a unique cytogenetic or a molecular defect, which might have been a potential therapeutic target. Instead, several factors are involved in disease development, such as environmental signals which interact with genetic abnormalities to promote survival, proliferation and an immune surveillance escape. Among these, PI3-Kinase signal pathway alterations are nowadays considered to be clearly important. The *TCL1* gene, an AKT co-activator, is the cause of a mature T-cell leukemia, as well as being highly expressed in all B-CLL. A *TCL1* transgenic mouse which reproduces leukemia with a distinct immunophenotype and similar to the course of the human B-CLL was developed several years ago and is widely used by many groups. This is a review of the CLL biology arising from work of many independent investigators who have used *TCL1* transgenic mouse model focusing on pathogenetic, microenviroment and therapeutic targets.

## Facts

- Aggressive form chronic lymphocytic leukemia (CLL) is still incurable.
- *The TCL1*-tg mouse model is most similar to aggressive human CLL.
- *The TCL1*-tg model has fundamentally contributed to the elucidation of CLL pathogenic mechanisms.
- Many novel therapeutic strategies have been tested using *TCL1*-tg mice.

## Open Questions

- Can new combination therapies be investigated using the *TCL1*-tg model?
- Can microenvironment contribution and tumor immune suppression be more easily studied through animal models than in human patients?
- Can microRNAs targeting *TCL1* or TCL1-specific inhibitors be used as therapies against CLL?

CLL is the most common B-cell malignancy in Western countries. CLL lymphocytes are similar to memory B-cells bearing a mature immunophenotype and showing different activation and maturation states.^1^ CLL patients manifest distinct disease courses^2, 3^ and prognostic molecular markers identify patients at different risk: leukemic clones with few *IgHV*-gene mutations (U-CLL) but with many CD38+ or ZAP70+ B-cells, lead to an aggressive disease, chemotherapy resistance and is usually fatal; clones with mutated *IgHV* (M-CLL), few CD38+ or ZAP70+ B-cells, exhibit an indolent asymptomatic course which generally responds to therapy.^4^ The monoclonal nature of leukemic cells suggests the existence of genetic lesions in the CLL. Recurrent cytogenetic aberrations include: deletion at 13q14.3 (55% of cases) is associated with an indolent form and loss of *miR-15a* and *miR-16-1* genes;^5^ deletions at 17p13 (7%) or 11q22-23 (18%) with consequent loss of *TP53* at 17p, *ATM* and *miR-34b/miR-34c* at 11q are associated with a more aggressive form;^6, 7^ trisomy 12 (16%) is associated with an intermediate form of CLL. Nucleotide sequencing has discovered recurrent mutations in a number of genes such as *TP53*, *NOTCH1*, *SF3B1*, *BIRC3* and *ATM*, which are indicative of derailed multiple pathways in CLL cells. It is also known that p53 mutations result in selective resistance to alkylating agents, such as fludarabine. In addition, treatment with DNA-damaging agents is correlated with an occurrence of p53 mutations in a clinical setting.^8^ Besides genetic lesions, pathogenic mechanisms may also represent survival signals arising from the microenvironment, through the B-cell receptor (BCR), integrins, chemokines and cytokine receptors, which allow CLL cells to actively proliferate and accumulate.^9, 10^

In addition, the T-cell leukemia-1 oncogene (*TCL1*) is expressed in almost all CLL patients and high-TCL1 protein levels correlate with the aggressive prognostic markers such as unmutated VH status, ZAP70 expression and chromosome 11q22-23 deletions.^11^ Accordingly, lower TCL1 levels are associated with a higher probability of positive response to chemoimmunotherapy.^12^ Animal models help to decipher pathogenic mechanisms of a disease and to evaluate the efficacy and mechanisms of novel therapies. A number of CLL mouse models have recently been reviewed by Simonetti *et al.*^13^ The authors compared different models and identified the Eμ-*TCL1* transgenic mouse (*TCL1*-tg) as the most similar to aggressive type human CLL, in terms of immunophenotype, BCR repertoire and disease course. Importantly, TCL1 overexpression exhibits a 100% disease penetrance.

## TCL1: Functions, Roles in CLL and Animal Models

**Figure 1:**
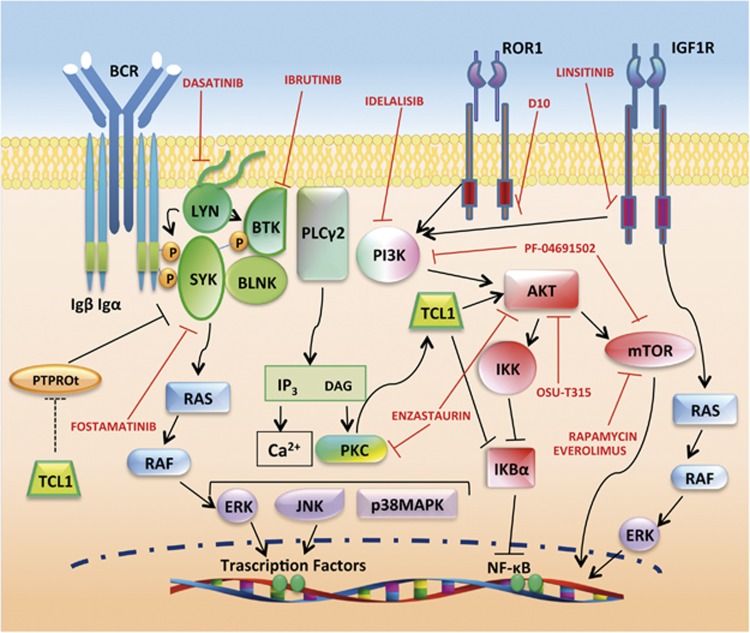
Targeting of BCR and PI3K/AKT signaling as therapeutic strategy in CLL. BCR signaling has a major role in the development of CLL. After antigen ligation on BCR, three main protein tyrosine kinases, LYN, SYK and BTK, are activated. PLC2 and PI3K are important downstream effectors of BCR signaling. PI3K activates downstream kinases such as AKT, which in turn induces NFkB and mTOR routes. Activation of PLC2 leads to the release of intracellular Ca2+ and activation of PKC, both of which are crucial for the activation of mitogen-activated protein kinases (MAPKs), such as ERK, c-JUN NH2-terminal kinase (JNK) and p38 MAPK and transcription factors, including NFκB. PI3K/AKT pathway can be induced also by tyrosine kinase receptors such as IGFR1 and ROR1. TCL1 enhances AKT signal and can sustain activation of the BCR downstream factors SYK and LYN by indirect inhibition of the phosphatase PTPROt. Red symbols and letters indicate new therapeutic targets as discussed in the text

**Figure 2:**
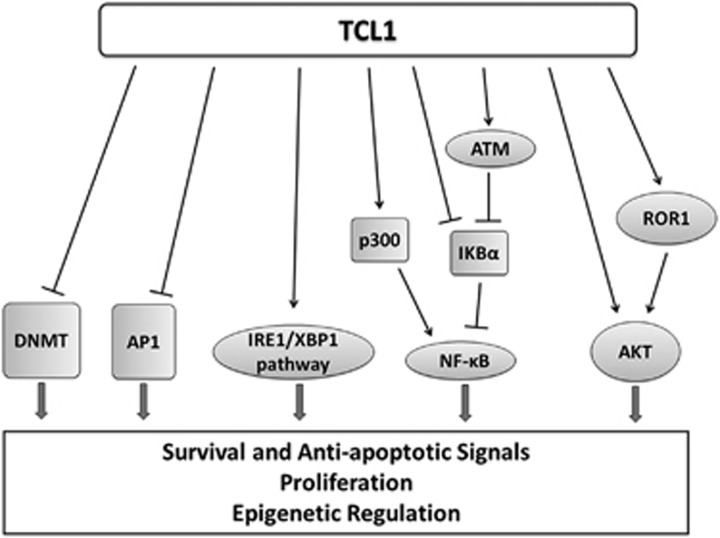
TCL1 function in CLL pathogenesis. TCL1 binds and regulates molecular factors implicated in proliferation, survival, inhibition of apoptosis and epigenetic regulation, thus contributing to CLL transformation

The *TCL1* gene was discovered as the causative oncogene of T-prolymphocytic leukemia (T-PLL), where it is overexpressed in almost 100% of cases by a chromosomal translocation.^14^ *TCL1* is also expressed in human seminomas,^15^ and in CD4+/CD56+ skin blastic tumors^16^ and in other B-cell lymphomas.^17^ TCL1 is a low-molecular weight protein and its first recognized function was the activation of phosphoinositide 3-kinase (PI3K) pathway, implicated in cell proliferation and survival (Figure 1), through direct binding with the AKT1/2 kinases.^18^ TCL1 binds to several other proteins and among these interacting proteins, the most relevant in CLL are: the receptor tyrosine kinase-like orphan receptor-1 (ROR1),^19^ the p300 transcription factor and the AP1 components FOS and JUN,^20^ the NFkB inhibitor alpha (IkB*α*),^21^ the XBP1 transcription factor^22^ and the DNA methyltransferases (DNMTs)^23, 24^ (Figure 2).

Physiologic functions of TCL1 protein mainly concern B-cell maturation, early embryonic development and stem cells regulation.^15, 17, 25, 26, 27^ Physiological roles of TCL1 was elucidated also by modifying *TCL1* expression in mice. The knocking out (KO) of *Tcl1* shows light impairment in B- and T-cell differentiation,^28^ while KO has stronger phenotypes in the embryonic stem cell proliferation/differentiation balance,^29^ embryo development^15^ and skin, especially in the hair follicle regeneration.^27^ This last KO phenotype is rescued when the strain is crossed to a *TCL1* transgenic mouse specific for epidermal basal layer, under the *Keratin14*-promoter.^27^

The overexpression of *TCL1* in transgenic animal models recapitulates faithfully leukemia of T-cell or B-cell origin according to the promoter used: the overexpression of *TCL1* in T cells under *Lck*-promoter, recapitulates human T-PLL^30^ and the overexpression of *TCL1* in B cells under the *V*_*H*_-promoter-*Ig*_*H*_-Eμ-enhancer (*TCL1*-tg), recapitulates CLL.^31, 32^ As in humans, leukemia developed in the *TCL1*-tg model is characterized by clonal expansion of B cells with B220+/IgM+/CD5+ immunophenotype, unmutated *IGHV*, increased proliferation and enhanced AKT phosphorylation, which represent an aggressive form of CLL.^31, 32, 33^ Leukemic cells are firstly detected in the peritoneal cavity, at 2 months age; then tumor cells become detectable in peripheral blood (PB) and expand to the spleen (at 4 months) and bone marrow (BM; at 8 months).^31^ Tumor cells in *TCL1* mice have wild-type (WT) p53 and initially respond to fludarabine treatment, after which drug resistance develops.^34^ Notably, the leukemic cells from a *TCL1*-tg donor can be transplanted by intra-peritoneum or intra-venous injection into syngeneic WT or immunodeficient mice (e.g., SCID) to accelerate the disease course and to generate a genetically homogeneous population of leukemic mice, which allows for the systematic study of novel therapies, without waiting for its natural course in non-transplanted animals. This technique can further be emphasized by serially adoptive cell transfer of leukemic cells into SCID mice. The repeated transplantations result in a clonal selection of B cells, which can be used for the analysis of particular conditions, for instance, BCR specificity.

## Therapeutic Targets in *TCL1*-tg Mouse Models

Nowadays, many studies have used these *TCL1*-tg mouse models (Table 1), and then contribute to our present knowledge of CLL biology and generate fundamental data for the development of new therapeutic approaches aimed at the overcome of drug resistance and the curative treatment of CLL. The following is a review of the data obtained from these mice models.

### PI3K/AKT pathway

As mentioned above, TCL1 directly binds to AKT (Figure 1) which enhances its phosphorylation and nuclear translocation.^18, 35^ AKT, originally isolated from leukemia and lymphoma-prone mice cells, is over expressed in many tumors and is a key factor in CLL, integrating survival and proliferative signals from the environment through BCR, growth factors, integrins, chemokines and TNF receptors.^36^ Oral administration of the AKT-inhibitor OSU-T315 in mice transplanted with *TCL1*-tg cells was shown to prolong survival^37^ and allowed for the elucidation of the drug mechanism acitivity: OSU-T315 displaces AKT from lipid rafts, thus impairing AKT activation regardless of activating pathways.

One of the AKT downstream factors is the protein kinase mammalian target of rapamycin (mTOR), which controls cell growth, proliferation and survival. Zanesi *et al.* established a syngenic transplantation model, where leukemic cells isolated from a *TCL1*-tg donor spleen can be indefinitely maintained *in vivo*. Treatment of transplanted mice with the mTOR inhibitor rapamycin slowed leukemia and prolonged survival.^38^

Recently, exciting results have been published using the dual PI3K/mTOR inhibitor PF-04691502 on the *TCL1*-tg.^39^ PF-04691502 is a potent antitumor agent able to inhibit all the PI3K isoforms and both the mTOR complexes 1 and 2 (mTORC1/2). This is necessary to overcome redundancy between PI3K isoforms and mTORC2 positive feedback on AKT phosphorylation, which were observed with the Food and Drug Administration (FDA)-approved specific inhibitors of PI3Kδ (idelalisib) and mTORC1 (everolimus). The study revealed the pro-apoptotic activity of PF-04691502 through caspase activation in both human and mouse CLL cells. Moreover, *in vivo* treatment of TCL1-tg mice allowed for further insight into the clinical effects of PF-04691502: inhibition of CXCL12-mediated migration toward spleen and lymph nodes (LNs) induced redistribution of the tumor cells from lymphoid organs to the blood, followed by a marked reduction of tumor burden due to the cytotoxic activity of the drug. The splenic architecture was maintained in treated mice, although tumor cells were not completely eradicated, reflecting some resistant subpopulation.

Alternatively, the AKT pathway can be affected through inhibition of upstream signals. For example, the insulin-like growth factor-1 receptor (IGF1R) is overexpressed in CLL and mediates IGF1-induced activation of PI3K/AKT and mitogen-activated protein kinase/extracellular signal-regulated kinase (ERK) pathways. Inhibition of IGF1R by oral administration of linsitinib in *TCL1*-tg mice produced a significant decrease in malignant cells.^40^

Also, AKT phosphorylation can be enhanced through overexpression of ROR1. *TCL1/ROR1* double-tg mice revealed the formation of complexes between the two factors and more aggressive leukemia due to increased proliferation and decreased apoptosis. *In vivo* administration of anti-ROR1 specific antibody, D10 revokes the potentiating effect of ROR1 on *TCL1*-tg cells, suggesting that this may be a novel therapeutic target in ROR1-expressing cancers.^19^

Finally, the overexpression of a member of the protein kinase C family, PKC*β*, involved in signal transduction of growth factors and BCR and known to be a PI3K-independent AKT activator, correlates with poor-prognosis in CLL patients. The cross talk between TCL1/AKT and PKC*β* was demonstrated in *TCL1*-tg mouse.^41^ In fact, genetic removal of PKC*β* prevents CLL development in crossed *TCL1*-tg/*Pkc*β^−/−^ mice. However, TCL1 overexpression restores AKT signaling and B-cells production, which were abrogated in parental *Pkcβ*^−/−^ mice, suggesting hierarchical order with TCL1/AKT acting downstream to PKC*β*. Besides the examination of the PKC/TCL1/AKT route in CLL, these results led to the *in vitro* evaluation of enzastaurin, which inhibits both PKC*β* and AKT, for therapeutic activity in CLL cells and subsequently to clinical trials (NCT00452257; Figure 1).

### Nuclear factor kappa-light-chain-enhancer of activated B cells (NFkB)

Anti-apoptotic activity of NFkB, mainly activated through BCR signaling, is an important factor in CLL etiology and several pieces of evidence indicate that TCL1 is involved in NFkB activation (Figure 2). TCL1 interacts with the p300 transcription factor, enhancing its ability to activate NFkB in human B cells.^20^ Also, TCL1 can directly interact with ATM and the NFkB inhibitor IkB*α*, thus enhancing the phosphorylation and degradation of IkB*α*, with consequent activation of NFkB both in human and mouse CLL.^21^ *In vivo* treatment with the inhibitor of chaperone protein HSP90 (17-DMAG or alvespimycin), depletes IkB kinase complex subunits (IKK) and inhibits NFkB transcriptional activity, resulting in reduced expression of anti-apoptotic proteins BCL2 and MCL1 and caspase-dependent apoptosis.^42^ The 17-DMAG is being tested in phase I clinical trials in CLL patients (NCT01126502).

### Endoplasmic reticulum (ER) stress response

ER stress response and IRE1/XBP1 pathway are aberrantly activated in human CLL and in *TCL1*-tg mice.^22^ TCL1 is directly involved in ER response by physical interaction with XBP1 and alteration of its transcriptional activity, resulting in constitutive activation of BCR signaling and influencing cross talk with other factors such as IRF4, BLIMP1 and AID. The importance of the IRE1/XBP1 pathway in leukemia maintenance is demonstrated by the observation that *in vivo* treatments with the specific inhibitor A-106 selectively induces apoptosis in *TCL1*-tg leukemic cells.^22^

On the other hand, prolonged activation of ER stress response by reactive oxygen species (ROS) may be exploited to induce cell death in CLL cells.^43^ Auranofin is a gold-containing drug, currently used in the rheumatoid arthritis treatment, which induces ROS levels. Oral administration of this FDA-approved compound to TCL1-tg mice markedly reduced leukemia expansion.^43^ Auranofin is currently investigated in phase II clinical trial for CLL therapy (NCT01419691).

### Epigenetic regulation

DNA methylation and histone modifications shape gene expression without changes in DNA sequences. Abnormalities affecting these epigenetic events are implicated in pathological conditions, including cancer and leukemia.

#### DNA methyltransferases

Methylation at cytosine residues of DNA is realized by DNMTs and causes repression of transcription. TCL1 appears to be directly implicated in such epigenetic regulation, although, diverse reports describe opposite roles. Chen *et al.* found increased methylation levels in human as well as mouse CLL cells^23^ and identified a repressor complex, constituted by TCL1, p50 subunit of NFkB and histone deacetylase 1 (HDAC1), which induces transcriptional silencing before DNA methylation. For example, transcriptional repression of the inhibitor of DNA-binding protein-4 (ID4) in *TCL1* mice results in acceleration of leukemia development.^44^ Conversely, Palamarchuk *et al.* described decreased methylation levels in *TCL1*-tg mice and in CLL patients.^24^ The authors found a strong interaction between TCL1 and *de novo* DNMT3A and 3B, with a drastic inhibition of enzymatic activity, suggesting a leukemogenesis mechanism involving inhibition of *de novo* methylation. However, Chen *et al.* observed the lack of DNMT3A and 3B in the genesis of the transformation of splenocytes from *TCL1*-tg mice, but increasing protein levels at later stages.^45^ Discrepancies in these data may reflect the complex timing of epigenetic changes with early event required for transformation and secondary events accumulating as a consequence of leukemogenesis. The analysis of the methylation status and transcriptional activation of these genes in *Tcl1*^−/−^ mice might provide additional clues on this issue.

#### Deacetylases

Deacetylases (DACs) are a family of enzymes, subdivided into classes I and II, which remove acetyl groups from a broad range of proteins. Histone proteins are the most studied target of DACs, but transcription factors, chaperones and signaling components are as much as important. Owing to the regulatory effects on cell growth and differentiation, inhibitors of DACs possess antitumor activity and are currently used in diverse solid cancers therapy. For example, HDAC inhibitors facilitate the formation of an active death-inducing signaling complex, leading to the rapid activation of caspase-8.^46^ Preclinical studies have also demonstrated the pro-apoptotic efficacy for class I-specific DACs inhibitors (romidepsin) in CLL cells and are currently in clinical trials. A novel class I/II DACs inhibitor, AR-42 (OSU-HDAC42) has been tested in a transplanted model of *TCL1*-tg mouse^47^ and demonstrated to be more potent than vorinostat, a pan-DAC inhibitor approved for cutaneous T-cell lymphoma therapy. AR-42 treatment in these mice resulted in diminished tumor growth and prolonged survival and this compound is now in phase I clinical trial (NCT01129193).

### MicroRNAs

MicroRNAs (miRNAs) are highly conserved small noncoding RNAs that function in post-transcriptional regulation. As miRNAs exhibit a variety of crucial functions related to cell growth, development and differentiation, dysregulation of miRNAs has been associated with diseases, including cancer. The first evidence for miRNAs involvement in human cancer was indeed from a study on CLL: 13q14.3 deletion, frequently observed in the patients, was shown to remove two microRNA genes, *miR-15* and *miR-16*; their loss causes deregulation of BCL2.^5, 48, 49, 50^ Subsequent studies, with the support of the *TCL1*-tg mouse model, disclosed an involvement of p53-miR15/16-MCL1 axis in the regulation of resistance.^51^ Loss of p53 in *TCL1*-tg/*p53*^−/−^ crossed mice causes a decrease of miR15/16 together with an upregulation of MCL1 and the consequent development of a more severe CLL. This association is fully consistent with the poor prognosis observed in CLL patients with the 17p deletion.

Extensive miRNA expression profiling on CLL cells from well-annotated cohorts of patients, identified miRNA signatures associated with prognosis and progression in B-CLL or with cytogenetic subgroups.^52, 53, 54^ Thus, inhibition of ‘oncomiRs' by anti-miRs or replacement of tumor suppressor miRNAs by mature sequence oligos (mimics) may represent novel therapeutic strategies. As CLL pathogenesis depends on several pathways and a single miRNA may have hundreds of mRNA targets, miRNA-based therapy might constitute a multibranched approach with higher potentialities than specific inhibitors.^55^

We have recently explored the therapeutic potential of miR-181b on the *TCL1*FL-tg mouse model.^32^ In addition, to its known function to modulate TCL1, MCL1 and BCL2,^11, 56, 57^ we observed the ability of this miRNA to downregulate important leukemia-promoting pathways such as AKT and ERK.^58^ Concordant with these findings, the treatment with miR-181b mimics reduces leukemic expansion and prolongs overall survival in *TCL1*FL-tg mice. This result represents the first *in vivo* demonstration for therapeutic efficacy of miRNA replacement strategy in CLL. Studies on additional miRNAs as well as combination with other conventional or innovative compounds might open new therapeutic possibilities, as suggested by the overcoming of resistance to the BCL2 inhibitors, through down-modulation of MCL1 and PI3K/AKT/mTOR pathway with siRNA or specific drugs.^59^

## B-cell Receptor

**Figure 3:**
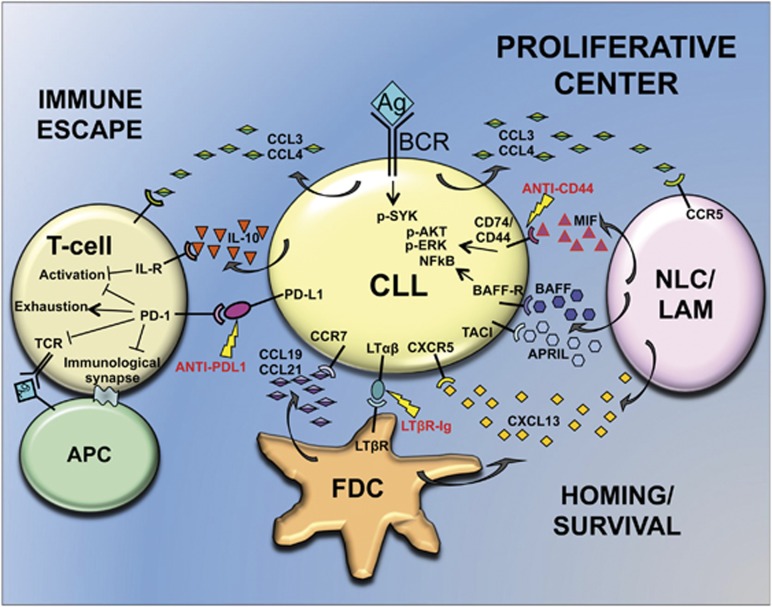
Leukemia-environment interplay within secondary lymphoid organs. A growth-promoting niche named proliferative center is the result of cross talk between tumor and accessory cells. CLL cells are attracted within LNs through chemokines (i.e., CXCL13 and CCL19/21) secreted by FDC and NLC, that are thought to correspond to leukemia-associated macrophages (LAM). Here, accessory cells provide a proliferative/survival milieu by secreting cytokines like MIF, BAFF and APRIL. CLL cells in turn, stimulate cytokine production by accessory cells, induce stromal cell differentiation through LT*αβ*/LT*β*R interaction and recruit NLCs and T cells through CCL3/4 secretion. An exhausted phenotype on T cells is also accomplished by CLL cells, through PD-L1/PD-1 interaction and IL-10 secretion to realize immune escape. Lightning symbols and red letters indicate new therapeutic targets as discussed in the text

CLL cells proliferate only in lymphoid organs, where accessory cells (i.e., nurse-like cells (NLC), T cells and stromal cells) provide the correct environment (mainly chemokines and cytokines) to sustain proliferation and survival of malignant cells^9^ (Figure 3). In this scenario, BCR signaling has a key role. A large amount of data, either from patients' studies or experimental models, supports the hypothesis of sustained antigen-dependent stimulation of BCR as a promoting event for clonal amplification of CLL cells.^1, 10, 60^ The sustained engagement of the BCR activates downstream targets such as NFkB, AKT and ERK,^61, 62^ which promote the expression of anti-apoptotic proteins, mainly BCL2 and MCL1.^36, 63^

BCR response is highly correlated with TCL1 levels in the CLL cells and with the formation of activation complexes at the BCR, by TCL1, AKT and ZAP70 kinases.^64^ Interplay between TCL1 and BCR activity was also suggested by studies on dn*Rag1*/*TCL1* double-tg.^65^ Enforced BCR auto-reactivity, due to RAG1 impairment, induces an indolent accumulation of CD5+ B cells, similar to monoclonal B-cell lymphocytosis; the *TCL1* overexpression provides an additional lesion on this background, promoting progression to CLL. Moreover, TCL1 can sustain activation of the BCR downstream factors spleen tyrosine kinase (SYK) and LYN, by inhibiting AP1-dependent transcription of the phosphatase, PTPROt.^20, 66^ PTPROt ability to regulate BCR signaling components have been established using the *TCL1/PTPROt* double-tg mouse,^67^ which exhibits decreased splenic cells growth and increased lifespan. For example, the chemokine CCL3, which is upregulated by BCR signaling, is repressed in double-tg respect to *TCL1*-tg mice.

CLL patients exhibit stereotyped BCRs, with unique HCDR3 features and recurrent *V*_*H*_*-D*_*H*_*-J*_*H*_ rearrangements, particularly in the *IGHV* unmutated cases.^68^ This observation led to the hypothesis that a subset of B-cells presenting stereotyped BCRs is selected by specific antigens such as auto antigens or microbial antigens and eventually these subsets get transformed by additional genetic abnormalities.^1^ *TCL1*-tg BCRs show HCDR3 characteristics and *V(D)J* rearrangements similar to U-CLLs.^33^ When B-lymphocytes reactive to auto antigen such as phosphatidylcholine (PtC) are serially transferred from *TCL1*-tg into SCID mice, a more aggressive leukemic clone is selected, showing increased reactivity with PtC over time. This finding is in agreement with an antigen selection and drive theory for leukemogenesis.^69^ An alternative hypothesis for BCR-induced CLL pathogenesis is proposed by Duhren-von Minden *et al*,^70^ who found an autonomous, ligand-independent BCR signaling in CLL samples from both human patients and *TCL1*-tg mice. This represents a new intriguing point of view, which does not exclude the hypothesis of extrinsic antigen involvement in CLL pathogenesis. Recently, the intrinsic/extrinsic types of BCR activation were examined in *TCL1* mice.^71^ CLL is supported by an aberrant auto antigen-driven response and BCR interactions are positively selected by low-affinity auto antigens during leukemia development. Thus, the two BCR responses might have independent roles with autonomous BCR activation being essential for disease initiation and the low-affinity interaction with external auto antigens providing powerful co-stimulatory signals.

Altogether these data sustain the importance of therapeutic approaches based on targeting BCR cascade. The close resemblance between BCRs from U-CLL patients and *TCL1*-tg mice validates the use of this model for preclinical studies to test the efficacy of inhibitors that block specific components of BCR pathways.

## Inhibitors of BCR Signalosome

Activation of BCR, either by extrinsic or intrinsic stimuli, transmits to membrane-associated signalosome proteins, composed of ‘proximal' kinases such as LYN, SYK, Bruton's tyrosine kinase (BTK), BLNK and PI3K. These, in turn, activate ‘distal' kinases, primarily ERK and AKT.^10^ Theoretically, each of the signalosome components might be a good candidate for targeted therapy, and increasing number of promising results have been achieved in this direction, some of which with the support of mouse models (Figure 1).

### Spleen tyrosine kinase

An extensive study on BCR signaling in *TCL1*-tg mice was performed by Suljagic *et al.* using SYK inhibitors.^72^ The authors found that SYK and its direct substrate BLNK are constitutively phosphorylated in some mice. In any case, BCR engagement led to the activation of downstream signals such as ERK, AKT, GSK3 and FOXO and this activation was reversed by the SYK inhibitor R406. Leukemic cells from *TCL1*-tg mice treated with R406 prodrug fostamatinib (R788) showed reduced phosphorylation at SYK, BLNK and ERK, decreased proliferation and increased apoptosis. Survival of treated mice was extended, leading in some cases to the eradication of malignant clones.^72^ Fostamatinib is currently tested in phase II clinical trial for B-cell lymphomas and CLL (NCT00446095).

### Bruton's tyrosine kinase

BTK inhibitor ibrutinib (PCI-32765) is one of the most recent FDA-approved drugs for refractory and aggressive 17p deleted form of CLL.^73^ XID mice bear a point mutation in *Btk* gene, which prevents its kinase activity.^74^ XID/*TCL1* crossed mice have lower tumor burden in PB and superior survival than *TCL1*-tg. Similarly to *Btk* genetic inactivation, ibrutinib treatment induced an increased overall survival in treated mice, which was shown to be dependent from the inhibition of BCR-induced ERK phosphorylation.^74^ *TCL1*-tg mouse also allowed for better understanding ibrutinib mechanism of action and observations from patients. For example, during a phase I–II clinical trial on CLL patients (NCT01105247) it was noted a transient increase in lymphocytosis, followed by rapid decline during the days of treatment;^75^ this effect is probably caused by ibrutinib-mediated block of chemokine signaling and secretion, that inhibits CLL cells homing in lymphoid organs, and it was also observed in Eμ-*TCL1* mice.^76^ Nevertheless, ibrutinib prolongs overall survival in these mice and induces significant decrease of CLL cells survival and proliferation demonstrating dual activity.^76^

### LYN kinase and hematopoietic cell-specific LYN substrate-1 (HS1)

HS1 is phosphorylated by SYK and LYN kinases on BCR engagement and its hyperphosphorylation correlates with a worst outcome in CLL patients.^77^ *TCL1*-tg/*Hs1*^−/−^ mice show accumulation of leukemic cells in all lymphoid tissues and shorter survival than *TCL1*-tg mice.^78^ As the genetic inactivation of *Hs1* produces the same effects of hyperphosphorylation, Scielzo *et al.* conclude that phosphorylation has an inhibitory effect on HS1. The therapeutic potential for targeting LYN and HS1 in CLL has been assessed in *TCL1*-tg transplantable mouse model.^79^ *In vitro* treatment with the tyrosine kinase inhibitor, dasatinib, prevents HS1 and ERK phosphorylation, induces apoptosis and blocks CXCL12 chemotaxis and the interaction with stromal cells. *In vivo* administration reduces LYN activity and the percentages of leukemic cells and delays CLL progression. Thus, dasatinib mechanism of action involves both cell survival and migration to specific tissues. Furthermore, the study highlighted the importance to analyze LYN/HS1 axis in CLL patients; in fact, dasatinib variable results in clinical trials^80^ may depend on LYN/HS1 activation status.^79^ Many clinical trials on dasatinib treatment in CLL are currently ongoing or have been completed (clinicaltrials.gov).

## Leukemia-Environment Interplay

Tumor microenvironment contributes to drug resistance and is crucial for the establishment of CLL proliferative centers, where malignant cells find optimal conditions to proliferate and survive. While environmental factors sustain tumor cells, tumor cells actively strive to establish a microenvironment in their favor, secreting chemokines that attract supportive cells and realizing complex strategies to escape immune surveillance.^9^ Thus, CLL-microenvironment interplay represents an interesting therapeutic target (Figure 3). The importance of microenvironment is also apparent in *TCL1*-tg as leukemic cells, obtained from a *TCL1*-tg donor and selected by serial transfer in SCID mice, display different response to BCR signaling based on cell residence:^69^ these cells actively proliferate in spleen and LN, but not in BM, peritoneal cavity and blood, despite their common origin (i.e., the spleen of the donor) and their clonal nature. Historically, BM has been considered the most important CLL microenvironment, where the stromal BM cells (SBMCs) secrete CXCL12 chemokine, and attract CXCR4-expressing CLL cells. However, increasing evidence support the relevance of LN. Gene expression analyses of leukemic cells in CLL patients, revealed a specific pattern of activation according to the tissue/organ source, such as PB, BM and LN.^81^ In particular, genes associated with signaling of BCR, BAFF/APRIL and some chemokines are overexpressed in LNs. High expression of phospho-SYK (BCR signaling) and phospho-p65 (NFkB pathway) are also found in LN from *TCL1*-tg mice.

Recently, Heinig *et al.* provided a clarifying dissection of the biological and molecular mechanisms underlying CLL cells homing to the LN.^82^ By crossing *TCL1*-tg with *Cxcr5*^−/−^ mice, these authors demonstrated this receptor to be indispensable for recruitment and proliferation of CLL cells into the germinal center mediated by CXCL13-expressing follicular dendritic cells (FDCs). In addition, *TCL1*-tg leukemic cells themselves are able to induce stromal cell differentiation through cell-bound lymphotoxin (lymphotoxin-*αβ* (LT*αβ*)). The proposed model is a recruitment of CLL cells by FDCs, via CXCL13/CXCR5 interaction, into a growth-promoting stromal niche, which provides BCR stimulation and paracrine cytokines (mainly BAFF). Reciprocally, leukemic cells induce stroma remodeling and CXCL13 secretion in a loop controlled by LT*αβ*. This mechanism offers at least two therapeutic targets: the CXCL13-CXCR5 axis and the LT*αβ* interaction with its receptor (LT*β*R), as demonstrated by *in vivo* treatment with an LT*β*R-Ig fusion protein, which abrogates the paracrine feedback loop between leukemic and stromal cells and retards leukemia growth.^82^

### Survival cytokines

Accessory cells in the leukemia microenvironment produce survival factors that inhibit spontaneous apoptosis. For example, BAFF and APRIL cytokines are highly expressed by NLCs. Overexpression of BAFF, by crossing *TCL1*-tg with *BAFF*-tg mice, induces faster development and more aggressive leukemia due to increased expression of anti-apoptotic proteins. The protective signal of BAFF is partly mediated by the NFkB pathway, also activated by TCL1.^83^ Similar results come from *TCL1/APRIL* double-tg.^84^ APRIL effects on CLL cells mainly rely on CD267/TACI TNF-receptor member and this is of therapeutic relevance as selective targeting of APRIL-TACI interaction may inhibit leukemic cells survival without affecting normal B-cells carrying another TNF-receptor member, namely CD269/BCMA.^84^

The macrophage migration inhibitory factor (MIF) is a proinflammatory cytokine acting on B cells, through the CD74/CD44 receptor complex. MIF loss in *TCL1*-tg/*Mif*^−/−^ mice delays CLL development and reduces leukemic cells survival.^85^ In addition, MIF can also act as a chemokine, recruiting M2 macrophages in leukemic organs, which, differently to cytotoxic type M1, promote tumor progression and suppress the immune response. These lymphoma-associated macrophages might correspond to NLCs.^85^ Further studies revealed a fundamental role for CD44 coreceptor. The *TCL1*-tg/*Cd44*^−/−^ crossed mice^86^ recapitulate the same phenotype of the *TCL1*-tg/*Mif*^−/−^ mouse model,^85^ exhibiting a reduced phosphorylation of major BCR downstream kinases (SYK, AKT and ERK) and reduced MCL1, no longer inducible by BCR engagement. As CD44 genetic deletion is mimicked by antibody-based targeting,^86^ MIF/CD44 signal pathway represents a potential target for therapy, which might be investigated in the *TCL1*-tg mouse model.

### Tumor immune suppression

Besides upregulation of survival and proliferative pathways by exploiting accessory cells, CLL cells induce also profound alteration of T-cell functions to realize tumor immune escape. Indeed, the rescue of immune surveillance by T cells is one of the greatest challenges for definitive CLL therapy. Immunosuppressive activity of CLL cells in the *TCL1*-tg mouse model has been well characterized and proved to be highly similar to human patients. Regulatory functions for CLL cells are supported by studies of Di Lillo *et al.*^87^ who showed that molecular mechanism underlying CLL-induced T-cell immunosuppression may be mediated by the cytokine IL-10. The competence of leukemic cells to express IL-10 either in human or mouse is normally found in B10 regulatory B cells, known to negatively regulate the immune response. In fact, mouse CLL cells suppress T-cell and monocyte/macrophage activation through IL-10-dependent pathways both *in vitro* and *in vivo*.

Gene expression profiling and protein expression analyses of CLL T-cells compared with WT or young *TCL1*-tg mice^88^ revealed that main changes involve proliferation, differentiation and cytokine/chemokine-response pathways, leading to functional impairment of antigen recognition, immune response, T-helper differentiation and cytotoxicity. Of note, using the transplantation technique a causal relationship between CLL, B-cells and T-cells changes has been found.^88^ Further rigorous transplantation experiments definitively demonstrated that CLL cells rapidly induce T-cell differentiation into memory compartment, probably through a tumor antigen-driven selection.^89^ Hence, T cells from human and TCL1-tg CLLs present a number of dysfunctions such as an impaired ability to form immunological synapse on conjugation with antigen-presenting cells, due to defects in cytoskeleton remodeling and in the recruitment of the T-cell receptor (TCR).^90^ The immunomodulatory drug lenalidomide is able to restore such defect and is extensively investigated in CLL clinical trials, especially in combination with other drugs (clinicaltrials.gov). Additional impairments in T-cell differentiation concern: the skewing from a naïve to an antigen-experienced memory compartment particularly in LNs,^89^ the reduction of CD4/CD8 ratios with the loss of central memory toward CD8+ effector memory pool,^91^ the exhausted phenotype characterized by poor effector function, the reduced cytokine production, the replicative senescence and finally the continued expression of inhibitory receptors.^92^ The latter is mainly represented by the receptors lymphocyte-activation gene 3 and programmed cell death 1 (PD-1) with PD-L1/2 ligands, which mediate dephosphorylation of signaling molecules downstream of the TCR. Actually, aberrant PD-1/PD-L1 signaling is involved in all the above mentioned T-cells dysfunctions.^91, 92^ In fact, normalization of the CD4/CD8 ratio, activation of T cells with restoring of effector cells cytotoxicity and immunological synapses formation, resolution of systemic inflammation and reversal of myeloid skewing are observed in the TCL1-tg mouse after anti-PD-1 antibody systemic treatment.^93^ Although a direct cytotoxic effect of the antibody on CLL cells was excluded, the treated mice showed a significant reduction of tumor load in disease-affected tissues, suggesting that PD-1/PD-L1 blockade is effective in tumor control restoration through immune effector functions.^93^ These remarkable results may represent an innovative strategy to decisively strengthen targeted therapy toward PI3K/mTOR or BCR signalosome inhibition.

Alternatively, to overcome the defective T-cell antitumor response, immunotherapy may be addressed to other cytotoxic effectors like macrophages.^94^ Treatment with CpG-containing oligodeoxynucleotides (CpG) synergizes with anti-CD40 mAb (*α*CD40) to activate macrophage antitumor response against human and mouse CLL cells, resulting in little or no tumor growth. *In vitro* analysis suggests that killing ability is partly mediated by nitric oxide synthesis. However, both human and mouse CLL cells express CD40 and toll-like receptor 9 (TLR9) and respond to treatment with *α*CD40 and CpG themselves, showing increased proliferation and modest protection from apoptosis. Thus, antitumor effects of activated macrophages must overcome the proliferative and anti-apoptotic effects of these stimuli on tumor cells.^94^ In agreement with this observation, the lack of TIR8 receptor which normally inhibits the signaling of TLRs, results in earlier and more aggressive CLL in *TCL1*-tg/*Tir8*^−/−^ crossed mice.^95^ Nevertheless, ligands for TLRs other than TLR9 might provide better and more specific activation of macrophages, resulting in improvement of CD40-based immunotherapy.

## Conclusions

*TCL1*-tg mouse model has been extensively investigated and the similarity with human U-CLL is striking. Thus, the *TCL1*-tg mouse represents a very useful model for a wide variety of studies, from basic molecular and cellular mechanisms, to fine dissection of the pathophysiology of CLL cells, including their interplay with tumor microenvironment and the preclinical evaluation of novel therapies. Further, a combination of two or more inhibitors, targeting pathways that cooperate to the insurgency, progression and relapse of CLL, is a topic that has never been tested in this mouse model and would be of considerable relevance for CLL therapy. TCL1 is not ubiquitously expressed being its expression, so far, limited to lymphoid, myeloid, cutaneous and embryonic cells.^15, 17, 25, 26, 27^ For this reason, therapies targeting TCL1 such as miRNAs or specific inhibitors of TCL1 protein in combination with pharmacological compounds currently used in the treatment of human B-CLL might represent also an interesting alternative to be tested on this animal model.

## Figures and Tables

**Table 1:** *TCL1*-tg animal models in B-CLL investigation

**Function**	**Mouse model**	**Findings**	**Relevance**	**Ref.**
*TCL*1 transgenic mouse models	TCL1-tg	TCL1 overexpression is causative for CLL	Mouse without UTRs of human TCL1	Bichi *et al.*^31^
	TCL1FL-tg	MicroRNAs regulation	Mouse with UTRs of human TCL1	Efanov *et al.*^32^
PI3K/AKT	TCL1-tg cells transplanted into C57bl/6 (i.v.)	AKT targeted therapy (OSU-T315)	Preclinical *in vitro* and *in vivo*	Liu *et al.*^37^
	TCL1-tg cells transplanted into B6/C3H (i.p.)	TCL1/AKT/mTOR pathway; mTOR targeted (rapamycin)	CLL pathogenesis; preclinical *in vivo*	Zanesi *et al.*^38^
	TCL1-tg cells transplanted into C57bl/6 (i.p.)	Dual PI3K/mTOR inhibitor (PF-04691502)	Preclinical *in vitro* and *in vivo*	Blunt *et al.*^39^
	TCL1-tg	Anti-IGFR1-targeted therapy (linsitinib)	Preclinical *in vitro* and *in vivo; CT*	Yaktapour *et al.*^40^
	TCL1-tg crossed with ROR-Tg	ROR1/TCL1 complex; anti-ROR1 Ab therapy (D10)	Preclinical *in vitro* and *in vivo*	Widhopf *et al.*^19^
	TCL1-tg crossed with Pkc null	PKC/TCL1/AKT route; PCK targeted (enzastaurin)	CLL pathogenesis; *CT*	Holler *et al.*^41^
NFkB	TCL1-tg	IkB*α*/TCL1 interaction and NFkB activation	Basic research; CLL pathogenesis	Guadio *et al.*^21^
	TCL1-tg cells transplanted into SCID (i.v.)	Anti-HSP90-targeted therapy (17-DMAG alvespimycin)	Preclinical *in vivo*; *CT*	Hertlein *et al.*^42^
	TCL1-tg	XBP1/TCL1 interaction, BCR signaling and IRE1/XBP1 targeted therapy (A-106)	Basic research; CLL pathogenesis; preclinical *in vitro* and *in vivo*	Kriss *et al.*^22^
	TCL1-tg	CLL therapy by ROS induction (Auranofin)	Preclinical *in vivo*; *CT*	Fiskus *et al.*^43^
Epigenetic regulation	TCL1-tg	TCL1/p50/HDAC1 complex and DNA methylation	Basic research; CLL pathogenesis	Chen *et al.*^23^
	TCL1-tg crossed with Id4^+/−^	ID4 repression and CLL progression	CLL etiology	Chen *et al.*^44^
	TCL1-tg	TCL1/DNMT3A-B interaction and DNA methylation	Basic research; CLL pathogenesis	Palamarchuk *et al.*^24^
	TCL1-tg	DNMT3A-B expression and leukemogenesis	CLL pathogenesis	Chen *et al.*^45^
	TCL1-tg cells transplanted into SCID (i.v.)	HDAC inhibition (AR-42)	Preclinical *in vivo*; *CT*	Lucas *et al.*^47^
	TCL1-tg crossed with p53 null	p53/miR15-16/Mcl1 axis	CLL pathogenesis	Liu *et al.*^51^
	TCL1-tg mice	MDM2/p53/miR34a axis	CLL pathogenesis	Asslaber *et al.*^96^
	TCL1FL-tg cells transplanted into FVB (i.p.)	miR-181b anti-leukemic activity	Preclinical *in vitro* and *in vivo*	Bresin *et al.*^58^
B-cell receptor	TCL1-tg crossed with dnRag1-Tg	TCL1 enhancement of BCR auto-reactivity	CLL pathogenesis	Nganga *et al.*^65^
	TCL1-tg	TCL1-induced PTPROt inhibition and BCR signal support	CLL pathogenesis	Motiwala *et al.*^66^
	TCL1-tg crossed with PTPROt-Tg	PTPROt overexpression and CLL phenotype rescue	CLL pathogenesis	Motiwala *et al.*^67^
	TCL1-tg	BCR resemblance to U-CLLs	CLL pathogenesis	Yan *et al.*^33^
	TCL1-tg cells serially transferred into SCID	Clonal selection and antigen drive	CLL pathogenesis	Chen *et al.*^69^
	TCL1-tg	Autonomous BCR signaling	CLL pathogenesis	Duhren-von minden *et al.*^70^
	TCL1-tg	Intrinsic/extrinsic BCR activation	CLL pathogenesis	Lacovelli *et al.*^71^
Inhibitors of BCR signalosome	TCL1-tg	SYK targeted therapy (fostamatinib R788)	CLL pathogenesis; preclinical *in vivo*; *CT*	Suljagic *et al.*^72^
	TCL1-tg crossed with XID	Btk inactivation and CLL pathogenesis	CLL pathogenesis	Woyach *et al.*^74^
	TCL1-tg	BTK targeted therapy (ibrutinib PCI-32765)	Preclinical *in vivo*; *CT*	Woyach *et al.*^74^
	TCL1-tg cells serially transferred into SCID	Ibrutinib mechanism of action	Preclinical *in vivo*; *CT*	Ponader *et al.*^76^
	TCL1-tg crossed with Hs1 null	Hs1 inactivation and CLL progression	CLL pathogenesis	Scielzo *et al.*^78^
	TCL1-tg cells transplanted into C57bl/6 (i.p.)	Tyrosine kinase inhibitor mechanism of action (dasatinib)	Preclinical *in vitro* and *in vivo; CT*	ten Hacken *et al.*^79^
Leukemia-environment interplay	TCL1-tg cells serially transferred into SCID	CLL cells proliferation in LNs	CLL pathogenesis	Chen *et al.*^69^
	TCL1-tg	BCR signaling activation in LNs	CLL pathogenesis	Mittal *et al.*^81^
	TCL1-tg crossed with Cxcr5 null	CXCL13/CXCR5 axis and CLL cells proliferation into LNs	CLL pathogenesis	Heinig *et al.*^82^
	TCL1-tg	Stroma remodeling by CLL cells; LT*β*R targeted (LT*β*R-Ig)	CLL pathogenesis; preclinical *in vivo*	Heinig *et al.*^82^
	TCL1-tg crossed with BAFF-Tg	BAFF/NFkB activation and CLL progression	CLL pathogenesis	Enzler *et al.*^83^
	TCL1-tg crossed with APRIL-Tg	APRIL/TNFR activation and CLL progression	CLL pathogenesis	Lascano *et al.*^84^
	TCL1-tg crossed with Mif null	MIF and activation of CLL cells and M2 macrophages	CLL pathogenesis	Reinart *et al.*^85^
	TCL1-tg crossed with Cd44 null	MIF/CD44 interaction and BCR signaling	CLL pathogenesis	Fedorchenko *et al.*^86^
	TCL1-tg	CD44 antibody-targeting (IM7)	Preclinical *in vitro* and *in vivo*	Fedorchenko *et al.*^86^
	TCL1-tg	IL-10-mediated immunosuppression by CLL cells	CLL pathogenesis	Di Lillo *et al.*^87^
	TCL1-tg and transplants into young TCL1-tg (i.v.)	T-cells expression profiling and tumor immune response	CLL pathogenesis	Gorgun *et al.*^88^
	TCL1-tg and TCL1-tg cells transferred into C57bl/6 (i.p.)	T-cells skewing by CLL cells	CLL pathogenesis	Hofbauer *et al.*^89^
	TCL1-tg	T-cells synapse and immunomodulation (lenalidomide)	CLL pathogenesis; preclinical *in vivo*; *CT*	Ramsay *et al.*^90^
	TCL1-tg and transplants into young TCL1-tg (i.v.)	T-cells defects and PD-1/PD-L1 expression	CLL pathogenesis	McClanahan *et al.*^93^
	TCL1-tg and TCL1-tg cells transferred into C57bl/6 (i.p.)	T-cells exhaustion and PD-1/PD-L1 blockade	CLL pathogenesis	Gassner *et al.*^92^
	TCL1-tg cells transplanted into C57bl/6 (i.p.)	T-cell immune response rescue by PD-1/PD-L1 blockade	CLL pathogenesis; preclinical *in vivo*	McClanahan *et al.*^91^
	TCL1-tg	Macrophages activation (*α*CD40 and CpG)	CLL pathogenesis; preclinical *in vivo*	Wu *et al.*^94^
	TCL1-tg crossed with Tir8 null	Tir8 inactivation and CLL progression	CLL pathogenesis	Bertilaccio *et al.*^95^

CT, clinical trials; LNs, lymph nodes; UTRs, untranslated regions.

## Abbreviations

CLL: chronic lymphocytic leukemia
TCL1: T-cell leukemia/lymphoma-1
T-PLL: T-prolymphocytic leukemia
KO: Knock out
WT: wild type
FDA: Food and Drug Administration
BCR: B-cell receptor
PI3K: phosphoinositide 3-kinase
mTOR: mammalian target of rapamycin
ER: Endoplasmic reticulum
NFkB: nuclear factor kappa-light-chain-enhancer of activated B cells
miR: miRNA microRNA
BTK: Bruton's tyrosine kinase
SYK: spleen tyrosine kinase
LN: lymph node
BM: bone marrow
PB: peripheral blood
FDCs: follicular dendritic cells
LT*αβ*: lymphotoxin-*αβ*
PD-1: programmed cell death 1
ERK: extracellular signal-regulated kinase
MIF: macrophage migration inhibitory factor
PtC: phosphatidylcholine
ROR1: receptor tyrosine kinase-like orphan receptor-1
